# Elaboration and validation of an algorithm for treating peripheral intravenous infiltration and extravasation in children

**DOI:** 10.1590/1518-8345.4314.3435

**Published:** 2021-06-28

**Authors:** Luciano Marques dos Santos, Katharinne de Jesus Nunes, Cleonara Sousa Gomes e Silva, Denise Miyuki Kusahara, Elisa da Conceição Rodrigues, Ariane Ferreira Machado Avelar

**Affiliations:** 1 Universidade Estadual de Feira de Santana, Departamento de Saúde, Feira de Santana, BA, Brazil.; 2 Universidade Federal de São Paulo, Escola Paulista de Enfermagem, São Paulo, SP, Brazil.; 3 Universidade Federal do Rio de Janeiro, Escola de Enfermagem Anna Nery, Rio de Janeiro, RJ, Brazil.

**Keywords:** Peripheral Catheterizations, Hospitalized Child, Pediatric Nursing, Extravasation of Diagnostic and Therapeutic Materials, Intravenous Infusions, Adverse Effects, Cateterismo Periférico, Criança Hospitalizada, Enfermagem Pediátrica, Extravasamento de Materiais Terapêuticos e Diagnósticos, Infusões Intravenosas, Efeitos Adversos, Cateterismo Periférico, Niño Hospitalizado, Enfermería Pediátrica, Extravasación de Materiales Terapéuticos y Diagnósticos, Infusiones Intravenosas, Efectos Adversos

## Abstract

**Objective:**

to elaborate and validate the content and appearance of an algorithm for treating infiltration and extravasation of non-chemotherapy drugs and solutions administered to children.

**Method:**

a methodological study of the technology formulation and validation type. To elaborate the algorithm, a bibliographic review was carried out to list the scientific evidence on the treatment of infiltration and extravasation. Content and appearance validation was in charge of 14 specialists in pediatric nursing, using the Delphi technique, adopting a value equal to or greater than 0.80 as Content Validation Index.

**Results:**

the algorithm was validated in the third evaluation by the judges, reaching a Global Content Validation Index of 0.99, being composed by the perception of the occurrence of the complication; discontinuation of intravenous therapy infusion; verification of signs and symptoms; measurement of edema; application of an infiltration and extravasation assessment scale and conduits to be used according to the characteristics of the fluid administered and the type of complication.

**Conclusion:**

the algorithm was validated and can be used in a practical and objective way by health professionals, in order to promote safety in the care of hospitalized children, with regard to reducing harms caused by infiltration and extravasation.

## Abstract (pt)

**Objetivo:**

elaborar e validar o conteúdo e a aparência de um algoritmo para tratamento de infiltração e extravasamento de medicamentos não quimioterápicos e soluções administradas em crianças.

**Método:**

estudo metodológico do tipo elaboração e validação de tecnologia. Para elaboração do algoritmo, realizou-se revisão bibliográfica para elencar as evidências científicas sobre o tratamento de infiltração e extravasamento. A validação do conteúdo e aparência ocorreu com 14 especialistas em enfermagem pediátrica, a partir da técnica Delphi, adotando-se como Índice de Validação de Conteúdo desejável os valores iguais ou superiores a 0,80.

**Resultados:**

o algoritmo foi validado na terceira avaliação pelos juízes, atingindo Índice de Validação de Conteúdo Global de 0,99, sendo composto por percepção da ocorrência da complicação; descontinuação da infusão da terapia intravenosa; verificação dos sinais e sintomas; mensuração do edema; aplicação de escala de avaliação de infiltração e extravasamento e condutas a serem utilizadas mediante a característica do fluido administrado e tipo de complicação.

**Conclusão:**

o algoritmo foi validado e pode ser usado de maneira prática e objetiva pelos profissionais de saúde, com o intuito de promover a segurança no cuidado da criança hospitalizada, no que tange à redução de danos provocados pela infiltração e extravasamento.

## Abstract (es)

**Objetivo:**

desarrollar y validar el contenido y la apariencia de un algoritmo para el tratamiento de la infiltración y extravasación de medicamentos no quimioterápicos y soluciones administrados a niños.

**Método:**

estudio metodológico de formulación y validación de tecnología. Para la elaboración del algoritmo, se realizó una revisión de la literatura para enumerar la evidencia científica sobre el tratamiento de la infiltración y extravasación. La validación de contenido y apariencia se dio con 14 especialistas en enfermería pediátrica, utilizando la técnica Delphi, adoptando como Índice de Validación de Contenido deseable valores iguales o superiores a 0,80.

**Resultados:**

el algoritmo fue validado en la tercera evaluación por los jueces, alcanzando un Índice de Validación de Contenido Global de 0,99, compuesto por la percepción de la existencia de la complicación; interrupción de la infusión de terapia intravenosa; verificación de signos y síntomas; medición del edema; aplicación de escala de evaluación de infiltración y extravasación y conducta según las características del líquido administrado y el tipo de complicación.

**Conclusión:**

el algoritmo ha sido validado y puede ser utilizado de forma práctica y objetiva por los profesionales de la salud, con el fin de promover la seguridad en la atención de los niños hospitalizados, en lo que respecta a la reducción de los daños causados por infiltración y extravasación.

## Introduction

Peripheral intravenous catheterization is an invasive procedure and is commonly performed in pediatric units^(^ 1 ^)^ for the administration of drugs, solutions, nutrients and blood products. Many of these fluids can cause local complications associated with intravenous therapy (IVT), defined as adverse events that cause signs and symptoms around the catheter insertion site^(^ 2 ^)^.

An international research study^(^ 3 ^)^ conducted with children from 278 hospitals in 47 countries in Africa, Asia, Australia/New Zealand, Europe, the Middle East, North America, South America and the South Pacific, demonstrated that, in 11.4% of the catheter insertion sites, signs of some complication were observed, while the incidence of these events was estimated at 18.6% in a prospective longitudinal study carried out in Bahia, Brazil^(^ 4 ^)^.

In the aforementioned research, carried out in Brazil, the following risk factors for complications associated with the use of peripheral IVT were identified: history of complications, prolonged use of this therapy, mainly non-irritating/vesicant drugs and vesicant solutions^(^ 4 ^)^.

It is noteworthy that, although there are fluids suitable for administration in peripheral veins, drugs that have a hydrogen potential (pH) of less than 5 or greater than 9 are not suitable for infusion by this route^(^ 2 ^)^, as they may increase the risk of infiltration, extravasation and depletion of the venous network throughout the child’s hospitalization period, such as irritants and vesicants.

Infiltration is characterized by the exit of a non-vesicant, non-irritating or irritating solution, from the intravascular to the extravascular space^(^ 2 ^,^ 5 ^)^, while the vesicant fluids will cause extravasation. A number of research studies show that infiltration is more frequent in children receiving medications such as 10% glucose, ampicillin/sulbactam, vancomycin, high-concentration electrolytes and phenytoin^(^ 6 ^)^ and that acyclovir, antibiotics, norepinephrine, dopamine, sodium bicarbonate, sodium chloride, calcium gluconate, propofol, contrast, blood and total parenteral nutrition^(^ 7 ^)^ cause more extravasation.

Recent research studies show that the frequency of infiltration in children varies from 2.9% to 35.8%^(^ 6 ^,^ 8 ^-^ 9) and extravasation from 17.6% to 17.9%^(^ 10 ^-^ 11 ^)^. Depending on the amount of fluid that is displaced from the intravascular to the extravascular space, the infiltration may compress the tissue surrounding the vessel, causing pain and swelling at the site, cold and pale skin, reduced mobility of the affected limb, decreased blood flow and extravasation of the solution at the catheter insertion site^(^ 5 ^)^. In turn, vesicating fluids will cause bubbles and tissue necrosis^(^ 2 ^,^ 5 ^)^, which may progress to compartment syndrome or amputation of part of the affected limb^(^ 9 ^)^.

Therefore, given the diagnosis of infiltration or extravasation due to the use of non-chemotherapy drugs and intravenous solutions, it is necessary for pediatric nurses to be able to, promptly, start the treatment of these adverse events, through evidence-based care that can reduce potential local or systemic harms. However, a research study conducted in the United States^(^ 12 ^)^ with 147 children with infiltration, indicated that among the precautions for the management of these complications, the removal of the intravenous catheter, use of hot or cold compresses, elevation of the limb or a combination of these treatments stood out.

Thus, it is necessary to standardize the interventions in view of the identification of infiltration and extravasation, to develop valid tools based on scientific evidence that can guide the care sequence that the pediatric nurse should perform, with regard to the initial treatment of these adverse events, by reducing harms and promoting safe care for hospitalized children.

An example of these tools are the algorithms, which contain standardized actions and contribute to the improvement of the clinical practice by directing health professionals in their actions^(^ 13 ^)^. Thus, the use of algorithms, as they contain a sequence of clearly defined and interconnected interventions, can facilitate the clinical judgment of the pediatric nurse and her decision-making in the face of an infiltration and extravasation.

In addition, an integrative review^(^ 14 ^)^ concluded that the incorporation of tools such as care protocols in the daily clinical practice contributes to the prevention and reduction of the severity of infiltration in children. However, in Brazil, there is a gap in the production of Nursing knowledge about the treatment of infiltrations and extravasation^(^ 15 ^)^ of non-chemotherapy drugs or solutions in children and algorithms that can be widely used in the daily clinical practice.

Thus, this study aimed to elaborate and validate the content and appearance of an algorithm for treating infiltration and extravasation of non-chemotherapy drugs and solutions administered to children.

## Method

A methodological, descriptive and exploratory study, of the technology elaboration and validation type, carried out from January 2016 to June 2017, through two stages: technology construction and validation^(^ 15 ^-^ 16 ^)^, by pediatric nursing specialists.

In the technology construction stage, articles published in journals indexed in the Virtual Health Library, Medical Literature Analysis and Retrieval System Online (MEDLINE), Latin American and Caribbean Center on Health Sciences Information (LILACS) and Scopus were identified. The following descriptors in Portuguese were used for the search: *Enfermagem Pediátrica, Criança, Criança Hospitalizada, Cateterismo Periférico, Infusões Intravenosas, Efeitos Adversos, Extravasamento de Materiais Terapêuticos and Diagnósticos*. Similar descriptors were also considered in English, Spanish and expressions of the Medical Subject Headings (MESH). These descriptors were crossed with each other, using the Boolean operators “AND” and “OR”.

The articles selected were those published in Portuguese, English or Spanish, between 2009 and 2016, available in full and that addressed interventions performed in the face of infiltration or extravasation in children. Editorials and letters to authors or editors were excluded.

The practice standards of the American Infusion Nurses Society (INS)^(^ 5 ^)^, INS Brazil^(^ 2 ^)^ and a book on infusion therapy were also consulted^(^ 17 ^)^. The selected articles were read in full, as well as the practice standards and the aforementioned book, and information was extracted regarding the interventions used during the treatment of infiltrations and extravasations.

*Microsoft Office Word and Adobe Acrobat Reader DC* were used to structure the algorithm. The first version of the algorithm consisted of a header, algorithm and references, distributed in five pages and entitled “Algorithm for treating peripheral intravenous infiltration and extravasation of non-chemotherapeutic drugs and solutions administered to children”.

Therefore, the algorithm was submitted to the content and appearance validation stage, from February to June 2017, using the *Delphi* technique. This technique was used due to the ease for obtaining the data, making it possible to carry out several evaluations until reaching consensus; accessibility to specialists in the theme of different regions, eliminating geographical limitations; reduction of induced answers, when collected in person. However, this technique is limited by the low adherence of the participants, delay in obtaining the data, and difficulty in selecting those with affinity for the theme^(^ 18 ^)^.

The study sample was of the intentional non-probabilistic type and no sample calculation was performed. Initially, possible participants were consulted by analyzing the resumes identified in the Lattes Platform, using the expression “intravenous infiltration in children” as keyword of the production. 38 potential resumes were identified, which, after analyzing the scientific production, allowed for the selection of 22 participants.

The criteria for the selection of participants were delimited by the researchers’ own experience in the subject matter, with no literature requirements being adopted. The following criteria were considered: being a health professional working in reference hospitals in the pediatric area as a service manager or direct assistance to children or in national public higher education institutions, involved in teaching and research; having a minimum experience of one year in the insertion of peripheral venous catheters in hospitalized children and treatment of local IVT complications. These last two criteria were validated after the participants’ acceptance. Those who did not complete all the stages of technology validation and those who did not fully answer the assessment instrument were excluded.

The 22 potential participants were invited via email, e-mail messages, obtaining consent from 14 participants and, of these, 13 remained until the third assessment. According to studies of algorithm validation, the number of participants varies between 19 and 38^(^ 15 ^-^ 16 ^,^ 19 ^-^ 20). However, it is verified in the literature that there is no consensus regarding the number of participants to make up the panel of experts^(^ 21 ^)^.

For the invitation, a letter was sent, containing the research objective, origin of the technology and method of validation, as well as the Free and Informed Consent Form. After the participants’ acceptance, the assessment instrument and the first version of the algorithm were sent.

The evaluation questionnaire was prepared by the researchers of this study, according to the assessment instrument of other studies regarding the validation of technologies^(^ 22 ^-^ 23 ^)^, presenting 14 questions considered relevant for the evaluation of the material regarding general impressions about the algorithm (four items), layout (four items), content (three items), motivation (two items) and applicability (one item), with opinions expressed through the “strongly disagree”, “disagree”, “agree”, “strongly agree” and “I don’t know” options, in addition to a space for reporting suggestions for technology adjustments. It also had information regarding the characterization of the judges.

The algorithm was validated after three evaluations and, in the first, the experts judged the technology according to the items presented in the validation instrument and commented on improvements to be made, being grouped in a chart according to the items, similarity and repetition. The suggestions were accepted as appropriate.

Therefore, the second evaluation was started with adjustments made to the algorithm, according to the suggestions that were accepted, only the variables that did not obtain the desirable level of agreement being assessed. Likewise, the suggestions from the second evaluation were assessed and the necessary adjustments were made to the algorithm, proceeding to the third evaluation, thus reaching validity.

Data was collected and entered twice using the Statistical Package for the Social Sciences (SPSS) software, version 22.0, the absolute and relative frequencies being analyzed for the characteristics of the panel of judges, in addition to mean, standard deviation, minimum and maximum. For the analysis, the Content Validity Index (CVI) of the category and global item was verified.

The CVI was calculated by dividing the total number of “strongly agree” plus “agree” answers by the total number of participants, being performed for the indexes of each item in the categories. The category CVI referred to the mean CVI of the specific items in each category. Finally, the Global CVI was carried out, with the relationship between the item CVI of all evaluated items divided by the total number^(^ 24 ^)^. For content validity of the technology, a CVI value of 0.80 or greater was considered as a desirable index.

The research was approved by the Research Ethics Committee of the State University of Feira de Santana, State of Bahia (BA), Brazil (Certificate of Presentation for Ethical Appreciation – CPEA number 34172014.7.0000.0053 and opinion 841612).

## Results

In the bibliographic survey stage, 17 productions were identified on the treatment of infiltration and extravasation, with only one of the articles presenting an algorithm for the treatment of extravasation of chemotherapeutic drugs in children with cancer.

The algorithm entitled “Algorithm for treating peripheral intravenous infiltration and extravasation of non-chemotherapeutic drugs and solutions administered to children” displays the following sequence of information provision: perception of the occurrence of the complication, discontinuation of the intravenous therapy infusion, verification of signs and symptoms, measurement of edema, application of an infiltration and extravasation assessment scale and conducts to be used according to the characteristic of the fluid administered and type of complication.

**Figure 1:**
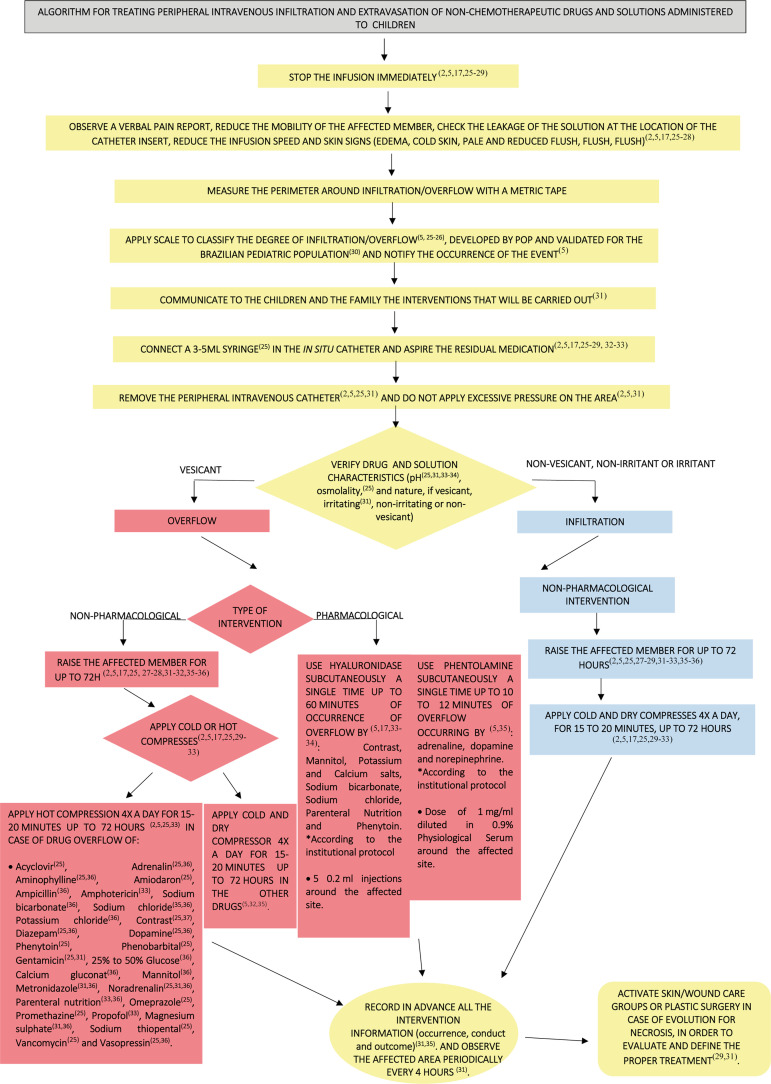
Final version of the “Algorithm for treating peripheral intravenous infiltration and extravasation of non-chemotherapeutic drugs and solutions administered to children”. Feira de Santana, BA, Brazil, 2016-2017

The treatment presented for extravasation was divided into two types: non-pharmacological and pharmacological, shown in Figure 1.

After the process of elaborating the algorithm, it was submitted to the evaluation of a group of specialists composed of 14 professionals, all of whom are nurses, female (92.9%), with PhD academic degrees (35.7%) and who work in their greatest proportion in the field of teaching and research (35.7%). Most work in an intensive care unit (26.9%), and for this variable it was possible to indicate more than one option.

The mean age of the specialists was 39.5 (± 9.03) years old, their professional training time was 15.2 (± 9.53) years, and the minimum time of professional training observed among the specialists was 5 years with a maximum of 36. The mean time of work in the pediatric area was 14.4 (± 10.5) years, with a minimum time in the area of one and a maximum of 36 years.

For the algorithm validation process, the Delphi technique was used, in which, by the evaluation method, it is necessary to reach the level of agreement. Thus, three evaluations were necessary, obtaining a Global CVI of 0.99. Tables 1, 2 and 3 show the CVI values for each technology assessment.

**Table 1:** Distribution of the Content and Appearance Validity Indexes given by the specialists (n=14) according to the content and language criteria of the first assessment. Feira de Santana, BA, Brazil, 2016-2017

Variables	CVI*
**General impressions about the algorithm**	
It is easy to use	0.92
It is self-explanatory	0.71
It is didactic	0.93
I recommend the algorithm for the practice of professionals dealing with intravenous therapy	0.92
**Category CVI**	**0.87**
**Layout**	
The visual composition is attractive and well organized	0.78
The way the information is arranged favors the learning of the theme	0.85
The instrument is easy to read	0.85
The colors used in the algorithm are pertinent	0.64
**Category CVI**	**0.78**
**Content**	
The content is scientifically correct	0.78
The information is clear and concise	0.85
The content has a logical organization	0.57
**Category CVI**	**0.73**
**Motivation**	
Feeling motivated to use the algorithm	0.85
The use of this technology can optimize the Nursing professional's working time	1
**Category CVI**	**0.92**
**Applicability**	
The technology has practical applicability	0.92
**Category CVI**	**0.92**
**Global CVI**	**0.84**

* CVI = Content Validity Index

**Table 2:** Distribution of the Content and Appearance Validity Indexes given by the specialists (n=14) according to the content and language criteria of the second evaluation. Feira de Santana, BA, Brazil, 2016-2017

Variables	CVI*
**General impressions about the algorithm**	
It is easy to use	1.00
It is self-explanatory	0.92
It is didactic	1.00
I recommend the algorithm for the practice of professionals dealing with intravenous therapy	1.00
**Category CVI**	**0.98**
**Layout**	
The visual composition is attractive and well organized	0.85
The way the information is arranged favors the learning of the theme	1.00
The instrument is easy to read	1.00
The colors used in the algorithm are pertinent	0.78
**Category CVI**	**0.90**
**Content**	
The content is scientifically correct	0.92
The information is clear and concise	1.00
The content has a logical organization	1.00
**Category CVI**	**0.97**
**Motivation**	
Feeling motivated to use the algorithm	1.00
The use of this technology can optimize the Nursing professional's working time	1.00
**Category CVI**	**1.00**
**Applicability**	
The technology has practical applicability	1.00
**Category CVI**	**1.00**
**Global CVI**	**0.97**

* CVI = Content Validity Index

**Table 3:** Distribution of the Content and Appearance Validity Indexes given by the specialists (n=13) according to the content and language criteria of the third evaluation. Feira de Santana, BA, Brazil, 2016-2017

Variables	CVI*
**General impressions about the algorithm**	
It is easy to use	1.00
It is self-explanatory	0.92
It is didactic	1.00
I recommend the algorithm for the practice of professionals dealing with intravenous therapy	**1.00**
**Category CVI**	**0.98**
**Layout**	
The visual composition is attractive and well organized	1.00
The way the information is arranged favors the learning of the theme	1.00
The instrument is easy to read	1.00
The colors used in the algorithm are pertinent	1.00
**Category CVI**	**1.00**
**Content**	
The content is scientifically correct	1.00
The information is clear and concise	1.00
The content has a logical organization	1.00
**Category CVI**	**1.00**
**Motivation**	
Feeling motivated to use the algorithm	1.00
The use of this technology can optimize the Nursing professional's working time	1.00
**Category CVI**	**1.00**
Applicability	
The technology has practical applicability	1.00
**Category CVI**	**1.00**
**Global CVI**	**0.99**

* CVI = Content Validity Index

In the first evaluation, the experts expressed that the algorithm did not prove to be self-explanatory, the visual composition was not attractive and well organized, the colors used in the algorithm were not relevant, the content was not scientifically correct and the content did not show logical organization (Table 1).

Through these results in the first evaluation, the experts proposed suggestions regarding the content, namely: define the interval for assessing the complication site, detail the form and frequency of application of the pharmacological antidotes, highlight the recommendations of the institutional protocol, identify the infiltration and extravasation scale.

The following were also proposed: to characterize the compresses, add other pharmacological measures, include criteria for the use of pharmacological measures, introduce a technology presentation paragraph, point out the need to record the conducts performed in the medical record, list the characteristics that must be evaluated in the medications or infused solutions and specify the type of conduct for each type of extravasated drug.

A judge requested the use of randomized controlled clinical studies on the use of compresses; however, no publications were found on the theme and with this type of research, with only observational studies and case reports available.

The layout was revised regarding the colors and size of the charts. Regarding the logical presentation, it was verified that the “stop the infusion immediately” approach was the first intervention to identify the complication. Regarding language, spelling corrections were made and phrases were restructured to improve comprehension.

After the corrections, the algorithm proceeded to the second assessment (Table 2). The experts pointed out that the colors used in the algorithm were not relevant, with suggestions to use lighter colors and the font color was changed to black. Other suggestions regarding the content were about the inclusion of signs and symptoms of the complications, how to assess pain and clarifying the wording of some excerpts.

In the third evaluation, only the category “the colors used in the algorithm were relevant” was evaluated and only 13 experts answered the assessment, obtaining desirable indexes in all the categories (Table 3). This latest version of the algorithm was updated with five studies published in 2019 and 2020 and the title was changed to adapt to the technology proposal, in the first version, “Treatment of infiltration and extravasation of non-chemotherapy drugs administered to children” for “Algorithm for treatment of peripheral intravenous infiltration and extravasation of non-chemotherapy drugs and solutions administered to children”, with the consent of the judges, which did not change the content already validated.

Figure 1 shows the final version of the algorithm for the treatment of infiltration and extravasation of non-chemotherapy drugs and solutions administered to children.

## Discussion

The “Algorithm for treating peripheral intravenous infiltration and extravasation of non-chemotherapeutic drugs and solutions administered to children” algorithm reached content validity in the third evaluation, together with specialists in the theme referring to IVT, with a Global CVI of 0.99.

An example of this, the algorithm directed to the treatment of extravasation of antineoplastic agents in children also reached content validity, through evaluation using the Delphi technique, with a rate higher than 80%, being analyzed by Brazilian and North American nurses^(^ 29 ^)^.

The development of this algorithm reflects a technology that addresses the treatment of infiltration and extravasation of non-chemotherapeutic drugs, given that there is an incipient production of these instruments for applicability in the clinical practice of pediatric nurses.

It is believed that the content validity of the algorithm allows for its use in care units for pediatric patients with need for IVT by the peripheral route because it is a technology that promotes adherence due to its accessibility and easy handling. As assessed by the experts, the algorithm was considered easy to use, being self-explanatory, didactic and recommended for the practice of professionals who deal with IVT.

In accordance with this result, in a national study on an algorithm for laser therapy in wounds, the evaluators demonstrated that the technology presented an excellent sequence, which is configured as didactic and self-explanatory^(^ 15 ^)^. Thus, these characteristics corroborate that the technology validated in this research is frequently used by pediatric nurses, as it provides rapid assessment and the use of appropriate interventions for the treatment of infiltration and extravasation in children.

As for the layout, the experts pointed out that the visual composition was attractive and well organized, that the way the information was arranged favored the learning of the theme, that the instrument was easy to read and that the colors used were relevant.

In a national survey on the construction and validation of an algorithm for cleaning and topical wound therapy, 30 nurses participated, 63.3% of whom considered the graphic presentation of technology to be excellent and 33.3%, good^(^ 16 ^)^. Regarding the validated algorithm, the questions regarding the visual structure are essential elements for its understanding and use, since it helps in directing the decisions in an objective way and provides the precise execution of the actions for the treatment, avoiding clinical worsening of the complication.

Regarding the content category, the experts stated that it was scientifically correct: the information was clear, concise and presented a logical organization. As described in the Method, the initial approach when detecting infiltration or extravasation is to stop the infusion^(^ 2 ^,^ 5 ^,^ 17 ^,^ 25 ^-^ 29 ^)^, aiming to decrease the amount of fluids in the region adjacent to the insertion area of the peripheral intravenous catheter, thus reducing potential harms. In addition, the clinical signs and symptoms of these two complications must be assessed^(^ 2 ^,^ 5 ^,^ 17 ^,^ 25 ^-^ 28).

The algorithm also evaluated the clinical severity of the complication based on a scale adapted for children and called “*Pediatric PIV Infiltration Scale*”, recently translated and adapted to Brazilian Portuguese^(^ 30 ^)^.

The aforementioned scale makes it possible to classify the infiltration in five grades. In grade 0, there are no symptoms characteristic of this complication and infusion flows easily. Grade 1 is identified by localized edema (1%-10%), difficulty in infusion and pain at the site. In grade 2, mild edema (up to ¼ or from 10% to 25% of the extremity above or below the insertion site), hyperemia and pain at the site can be identified. Regarding grade 3, moderate edema (¼ to ½, or 25%-50% of the extremity above or below the insertion site), pain at the site, cold skin to the touch, pallor at the site, and pulse decreased below the site can be observed; and, in grade 4, edema classified as severe (more than ½ or 50% of the extremity above or below the insertion site), cold skin to the touch, pallor at the site, skin rupture/necrosis, blistering, decreased or absent pulse, pain at the site and capillary filling > 4 seconds^(^ 30 ^)^.

In general, small volume infiltrations and extravasations with less potent vesicating fluids will be classified as stages 1 or 2, while more powerful vesicants and/or larger extravasated volumes tend to reach stages 3 or 4^(^ 33 ^)^. In this sense, the conducts for handling infiltrations and extravasations can be different, considering the amount of fluid displaced and the grade of the edema.

In the algorithm, the importance of communicating with the family members and children is emphasized by providing information about the occurrence of the complication and clarifying the conducts that will be adopted^^(^31^)^^. The patients and their families must be informed about the extent of the lesion^(^ 26 ^)^. Thus, when providing this information, the pediatric nurse will promote Family and Child Centered Care, respecting the premises of dignity and respect, information sharing, participation and collaboration^(^ 38 ^)^.

According to a research study carried out with family members of hospitalized children in need of peripheral intravenous catheterization, it was verified that the provision of information about the procedure produces safety, given that the family members express concerns about the complications associated with the procedure and the possibility of performing a new catheterization. The family members also expressed that the health professionals must direct information about the procedure to their children^(^ 39 ^)^.

One of the first procedures to be performed with the *in situ* catheter is its aspiration through the connector or hub of the catheter to remove the largest volume of the liquid^(^ 2 ^,^ 5 ^,^ 17 ^,^ 25 ^-^ 29, 32-33) being infiltrated or extravasated.

Large volumes of infiltrated/extravasated and anatomically located fluids can cause vasoconstriction, by mechanical compression, when interstitial pressure is high enough to overcome venous pressure, blocking blood flow and, even, causing compartmental syndrome^(^ 33 ^)^. Therefore, it is important to raise the affected limb, as this can assist in the reabsorption of the infiltrated or extravasated fluid, contributing to the reduction in capillary hydrostatic pressure^(^ 26 ^)^.

The mechanism for the occurrence of infiltration and extravasation depends on the type of medication displaced to the extravascular space. Fluids that exhibit pH extremes (below 5 or over 9) irritate the vascular endothelium^(^ 33 ^-^ 34 ^)^, making the vessel more vulnerable to inflammation and rupture^(^ 34 ^)^. Medications and acid solutions cause vasoconstriction, edema, and disintegration of the skin, with evolution to necrosis caused by protein coagulation and can lead to the formation of an ulcer^(^ 31 ^)^.

Alkali, in turn, cause protein dissolution, collagen destruction and fatty acid saponification, leading to membrane rupture and cell death. After erythema and edema, the denaturation of the extracellular matrix allows for the diffusion in depth of the hydroxide ions, causing damage to the tissue, which can be similar to a liquefactive necrosis. The damage observed is generally worse than with acid agents^(^ 25 ^)^.

Hypertonic solutions cause water imbalance between the intracellular and extracellular compartments, causing fluid displacement, dysfunction and cell death. The accumulation of liquid also compromises the tissue through hypoperfusion and, subsequent, tissue necrosis^(^ 25 ^)^.

Vasoactive medications - they promote alpha receptor stimulation and constrict capillary beds^(^ 40 ^)^, reducing blood flow in peripheral vessels^(^ 31 ^,^ 33 ^,^ 40), leading to edema, inflammation^(^ 31 ^)^, severe tissue hypoxia and ischemia^(^ 31 ^,^ 40 ^)^.

Vasodilator medications, such as dobutamine and dopamine, increase the lesion resulting from extravasation, increasing the local blood flow and the lesion area. Electrolytes, like calcium, stimulate smooth muscles to contract the capillaries, leading to hypoperfusion and ischemic injury^(^ 41 ^)^.

Fat-soluble medications cause local harms by remaining in the tissue for a long time, hindering absorption due to low solubility^(^ 31 ^,^ 41 ^)^. Parenteral nutrition is a complex mixture of substances, including nitrogen, glucose, lipids, and electrolytes, potassium and calcium, vitamins and trace elements. It is hyperosmolar and its local tissue toxicity is due to a combination of toxic effects of the local ions, hyperosmolality and acid pH of the solution^(^ 41 ^)^.

Thus, the use of cold, hot and dry compresses, applied for 15 to 20 minutes every 4 hours, for 24 to 48 hours^(^ 2 ^,^ 5 ^,^ 17 ^,^ 25 ^,^ 29 ^-^ 33) is necessary to reduce the local reaction and the absorption of the infiltrated medication or solution^(^ 26 ^)^ and contribute to the reduction of potential injuries.

Cold and dry compresses are indicated in order to reduce the absorption of the extravasated fluid, keeping it localized and reducing the triggering of inflammatory processes. This type of compress is indicated for non-irritating and hyperosmolar medications and solutions^(^ 5 ^)^. In addition, the use of hot and dry compresses was indicated, in order to promote vasodilation and dispersion of the fluid through the tissues adjacent to the catheterization site^(^ 5 ^,^ 35 ^)^.

Regarding the pharmacological treatment, the use of hyaluronidase and phentolamine is highlighted^(^ 5 ^,^ 16 ^,^ 33 ^-^ 34). Hyaluronity is defined as an enzyme that acts by degrading hyaluronic acid, which acts by intensifying intercellular bonds and preventing the dispersion of the extravasated fluid; thus, the enzyme acts by breaking the connections and facilitating the absorption of the medications into the bloodstream^(^ 5 ^,^ 25 ^,^ 42).

A research study carried out with 13 cases of extravasation in neonates with the treatment of hyaluronidase showed that all children presented edema (100%), erythema (38.46%), blister (23.08%) and tissue necrosis (7.69%) as clinical signs edema, erythema, blisters and tissue necrosis^(^ 43 ^)^.

Phentolamine acts as an antagonist of the alpha-1 receptors located in the blood vessels in order to cause vasodilation and increase the absorption of vasoconstrictor drugs, preventing the occurrence of local necrosis due to poor blood circulation^(^ 25 ^)^.

Another treatment presented in a literature review was the use of saline irrigation with prior infiltration of hyaluronidase; however, for this type of action, effectiveness was not verified through rigorous studies of randomized clinical trials^(^ 44 ^)^. In addition, there is the combined therapy between hyaluronidase and hot compress, which has the adjuvant purpose of increasing the absorption of extravasated fluids with a consequent reduction in local harms^(^ 36 ^,^ 45 ^)^.

After conducting the procedures for the management of infiltration or extravasation, some information must be recorded: date and time of the extravasation, name of the extravasated medication, signs and symptoms, description of the extravasation area, approximate amount of extravasation and interventions performed. The photographic record can be useful for future assessments of the complication site and for monitoring its evolution^(^ 26 ^)^.

The algorithm demonstrates the need for injury monitoring by a group of wound specialists, with plastic surgery being indicated in case of tissue necrosis^(^ 29 ^,^ 31 ^)^. Subsequent assessments should be made based on whether the injury is receding or advancing^(^ 26 ^)^.

Due to the various possibilities of pharmacological and non-pharmacological treatments, there is still little evidence to support its use in a completely safe manner; thus suggesting the need to develop clinical and randomized trials to achieve more effective results in the clinical practice.

As for the motivation category, the evaluators judged that they felt motivated to use the algorithm and that this technology would optimize the Nursing professional’s working time. The question of practical applicability also obtained maximum satisfaction, which demonstrates the importance of developing and validating technologies applicable to the clinical context of pediatric nurses, strengthening the translation of knowledge and the implementation of scientific evidence in the care of hospitalized children, promoting patient safety.

In an international study, the results showed a reduction in cases of infiltration in children after the implementation of an educational project for nurses on the development of practices based on scientific evidence for the insertion and maintenance of intravenous devices via the peripheral route, in addition to having the collaboration of family members as protagonists in the process of early identification of the adverse event^(^ 46 ^)^.

A research study highlighted that, after the applicability of a guideline that defines the flow of care for children and newborns who had an extravasation to specialized care, there was a significant reduction in the occurrence of tissue necrosis^(^ 7 ^)^, which adds value to the use of the technology validated in this research, as a resource for the management of infiltrations and extravasations.

However, this research has some limitations. The content and appearance validation has a subjective nature, being necessary to verify the practical applicability of the proposed algorithm. In addition, the scarce production of knowledge about the construction and validation of algorithms for the treatment of infiltration and extravasation in children made it difficult to develop the discussion of the data presented.

However, the study presents theoretical, practical and social contributions. With regard to theory, it will be able to strengthen the curricular components that involve care for hospitalized children through the use of this visual technology in clinical Nursing and IVT teaching and thus promote critical reflections on child care, innovating undergraduate and graduate teaching.

For workers in the clinical practice, the instrument may direct child care, helping them to use interventions based on scientific evidence. In addition, children and their families can benefit from access to safe and adequate assistance to their needs, in addition to being able to minimize the suffering caused by the occurrence of infiltration and extravasation.

## Conclusion

The “Treating infiltration and extravasation of non-chemotherapy drugs and solutions administered to children” algorithm was developed according to a literature review and considered valid in terms of content for use in the clinical practice, according to an evaluation by specialists in the field of pediatrics.

The instrument is configured as a technology that can be used in a practical and objective way by health professionals, with the aim of promoting patient safety with regard to reducing the harms caused by the occurrence of infiltration and extravasation in children.
